# Supplementary material for: Understanding the global subnational migration patterns driven by hydrological intrusion exposure
Source: Nat Commun. 2024 Jul 26;15:6285. doi: 10.1038/s41467-024-49609-y (PMC11282214; doi:10.1038/s41467-024-49609-y)
Supplement: Supplementary file 1 — Supplementary Information [file 41467_2024_49609_MOESM1_ESM.pdf]

Supplementary Information

	Variable	Description	Mean	SD	Ref.
Exposure Variable	HIE (%)	This metric assesses the region's exposure level to hydrological risk.	1.35	2.30	
	HIE_D (%)	This metric measures the difference in HIE between neighboring areas.	-36.25	148.72	
Hazard Variable	Rx5day (mm)	Represents the highest precipitation received over five consecutive days within a year.	104.56	67.60	[1]
Vulnerability Variable	PRCP (mm)	General measure of rainfall, providing an overview of water availability and potential flood risk.	42.68	29.65	[2]
	Water_Dis (km)	The proximity of a location to rivers, lakes, or other water bodies.	87.92	101.09	[2]
	TEMP (℃)	Average temperature measurement.	16.66	8.20	[3]
	DEM (m)	A representation of a region's elevation.	388.53	545.16	[2]
	GDP_PA (Million dollars/km^2)	Economic productivity is measured, indicating regional economic strength and development.	6.06	15.18	[4]
	NTL_PA	Satellite imagery measuring artificial lighting at night.	2.31	8.60	[5]
	Urban_R (%)	The rate of urbanization.	17.16	22.58	[5]
	Edu (Years)	Average years of education of the population.	9.95	3.02	[3]

Supplementary Table. 1 The variables used in defining hydrological risk

Noted: This study employed a dataset that consists of eleven categories of variables and they were used to describe the hydrological risk in exposure, hazard, and vulnerability.

Region	ISO
Australia/New Zealand	AUS; CCK; CXR; HMD; NFK; NZL

Caribbean	ABW; AIA; ATG; BES; BHS; BLM; BRB; CUB; CUW; CYM; DMA; DOM; GLP; GRD; HTI; JAM; KNA; LCA; MAF; MSR; MTQ; PRI; SXM; TCA; TTO; VCT; VGB; VIR
Central America	BLZ; CRI; GTM; HND; MEX; NIC; PAN; SLV; XCL
Central Asia	KAZ; KGZ; TJK; TKM; UZB
Eastern Africa	BDI; COM; DJI; ERI; ETH; KEN; MDG; MOZ; MUS; MWI; MYT; REU; RWA; SOM; SSD; SYC; TZA; UGA; ZMB; ZWE
Eastern Asia	CHN; HKG; JPN; KOR; MAC; MNG; PRK; TWN; XPI; XSP
Eastern Europe	BGR; BLR; CZE; HUN; MDA; POL; ROU; RUS; SVK; UKR
Melanesia	FJI; NCL; PNG; SLB; VUT
Micronesia	FSM; GUM; KIR; MHL; MNP; NRU; PLW
Middle Africa	AGO; CAF; CMR; COD; COG; GAB; GNQ; STP; TCD
Northern Africa	DZA; EGY; ESH; LBY; MAR; SDN; TUN
Northern America	BMU; CAN; GRL; SPM; UMI; USA
Northern Europe	ALA; DNK; EST; FIN; FRO; GBR; GGY; IMN; IOT; IRL; ISL; JEY; LTU; LVA; NOR; SGS; SJM; SWE; BVT; PCN; XAD
Polynesia	ASM; COK; NIU; PYF; TKL; TON; TUV; WLF; WSM
South America	ARG; BOL; BRA; CHL; COL; ECU; FLK; GUF; GUY; PER; PRY; SUR; URY; VEN
South-Eastern Asia	BRN; IDN; KHM; LAO; MMR; MYS; PHL; SGP; THA; TLS; VNM
Southern Africa	BWA; LSO; NAM; SWZ; ZAF
Southern Asia	AFG; BGD; BTN; IND; IRN; LKA; MDV; NPL; PAK
Southern Europe	ALB; AND; BIH; ESP; GIB; GRC; HRV; ITA; MKD; MLT; MNE; PRT; SMR; SRB; SVN; VAT; XKO
Western Africa	BEN; BFA; CIV; CPV; GHA; GIN; GMB; GNB; LBR; MLI; MRT; NER; NGA; SEN; SHN; SLE; TGO
Western Asia	ARE; ARM; AZE; BHR; CYP; GEO; IRQ; ISR; JOR; KWT; LBN; OMN; PSE; QAT; SAU; SYR; TUR; YEM; XNC
Western Europe	ATF; AUT; BEL; CHE; DEU; FRA; LIE; LUX; MCO; NLD

Supplementary Table. 2 The list of countries

**Data**	**Source**
WorldPop	https://hub.worldpop.org/
Global surface water dynamics	https://glad.umd.edu/dataset/global-surface-water-dynamics
ERA5-Land	https://www.ecmwf.int/en/forecasts/dataset/ecmwf-reanalysis-v5
SRTM	https://www.earthdata.nasa.gov/sensors/srtm
NTL	https://dataverse.harvard.edu/dataset.xhtml?persistentId=doi:10.7910/DVN/YGIVCD
GDP	https://doi.org/10.1038/s41597-022-01322-5
SHDI	https://globaldatalab.org/shdi/

Supplementary Table. 3 Data sources

References

1 Zhang, W., Zhou, T., Zou, L., Zhang, L. & Chen, X. Reduced exposure to extreme precipitation from 0.5 °C less warming in global land monsoon regions. *Nature Communications* **9**, 3153 (2018). https://doi.org/10.1038/s41467-018-05633-3

2 Hauer, M. E. *et al.* Sea-level rise and human migration. *Nature Reviews Earth & Environment* **1**, 28-39 (2020). https://doi.org/10.1038/s43017-019-0002-9

3 Brottrager, M., Crespo Cuaresma, J., Kniveton, D. & Ali, S. H. Natural resources modulate the nexus between environmental shocks and human mobility. *Nature Communications* **14**, 1393 (2023). https://doi.org/10.1038/s41467-023-37074-y

4 Schutte, S., Vestby, J., Carling, J. & Buhaug, H. Climatic conditions are weak predictors of asylum migration. *Nature Communications* **12**, 2067 (2021). https://doi.org/10.1038/s41467-021-22255-4

5 Niva, V. *et al.* World's human migration patterns in 2000–2019 unveiled by high-resolution data. *Nature Human Behaviour* **7**, 2023-2037 (2023). https://doi.org/10.1038/s41562-023-01689-4
