# Supplementary material for: Understanding the global subnational migration patterns driven by hydrological intrusion exposure
Source: Nat Commun. 2024 Jul 26;15:6285. doi: 10.1038/s41467-024-49609-y (PMC11282214; doi:10.1038/s41467-024-49609-y)
Supplement: Supplementary file 3 — Reporting Summary [file 41467_2024_49609_MOESM3_ESM.pdf]

Corresponding author(s): Zhiyu Liu, Shiqi Zhou, Mo Wang, and Zhiqiang Wu
NCOMMS-23-57887A

Last updated by author(s): May 27, 2024

Reporting Summary

Nature Portfolio wishes to improve the reproducibility of the work that we publish. This form provides structure for consistency and transparency in reporting. For further information on Nature Portfolio policies, see our Editorial Policies and the Editorial Policy Checklist.

Statistics

For all statistical analyses, confirm that the following items are present in the figure legend, table legend, main text, or Methods section.

n/a	Confirmed	
☐	☒	The exact sample size (*n*) for each experimental group/condition, given as a discrete number and unit of measurement
☐	☒	A statement on whether measurements were taken from distinct samples or whether the same sample was measured repeatedly
☐	☒	The statistical test(s) used AND whether they are one- or two-sided<br>*Only common tests should be described solely by name; describe more complex techniques in the Methods section.*
☐	☒	A description of all covariates tested
☒	☐	A description of any assumptions or corrections, such as tests of normality and adjustment for multiple comparisons
☐	☒	A full description of the statistical parameters including central tendency (e.g. means) or other basic estimates (e.g. regression coefficient) AND variation (e.g. standard deviation) or associated estimates of uncertainty (e.g. confidence intervals)
☐	☒	For null hypothesis testing, the test statistic (e.g. *F*, *t*, *r*) with confidence intervals, effect sizes, degrees of freedom and *P* value noted<br>*Give P values as exact values whenever suitable.*
☒	☐	For Bayesian analysis, information on the choice of priors and Markov chain Monte Carlo settings
☐	☒	For hierarchical and complex designs, identification of the appropriate level for tests and full reporting of outcomes
☐	☒	Estimates of effect sizes (e.g. Cohen's *d*, Pearson's *r*), indicating how they were calculated

Our web collection on statistics for biologists contains articles on many of the points above.

Software and code

Policy information about availability of computer code

Data collection	No software was used.
Data analysis	We used ArcGIS Pro 2.5.0 and Python 3.8 to analyze the data. Code for the main modeling and analysis process is available on Zenodo at https://doi.org/10.5281/zenodo.10911389.

For manuscripts utilizing custom algorithms or software that are central to the research but not yet described in published literature, software must be made available to editors and reviewers. We strongly encourage code deposition in a community repository (e.g. GitHub). See the Nature Portfolio guidelines for submitting code & software for further information.

Data

Policy information about availability of data

All manuscripts must include a data availability statement. This statement should provide the following information, where applicable:

- Accession codes, unique identifiers, or web links for publicly available datasets
- A description of any restrictions on data availability
- For clinical datasets or third party data, please ensure that the statement adheres to our policy

Data relevant to this study can be downloaded from the following website: WorldPop, https://hub.worldpop.org/; Global surface water dynamics, https://glad.umd.edu/dataset/global-surface-water-dynamics; ERA5-Land, https://www.ecmwf.int/en/forecasts/dataset/ecmwf-reanalysis-v5; SRTM, https://www.earthdata.nasa.gov/sensors/srtm; NTL, https://dataverse.harvard.edu/dataset.xhtml?persistentId=doi:10.7910/DVN/YGIVCD; GDP, https://doi.org/10.1038/s41597-022-01322-5; SHDI, https://globaldatalab.org/shdi/.

Research involving human participants, their data, or biological material

Policy information about studies with human participants or human data. See also policy information about sex, gender (identity/presentation), and sexual orientation and race, ethnicity and racism.

Reporting on sex and gender	In the present study, the population was stratified into two groups based on sex, a biological attribute, to conduct distinct modeling analyses for each group. This methodological choice was informed by the recognition of potential differences in the variable of interest across sex groups. The study did not rely on individual-level data. Instead, it utilized raster data from WorldPop (https://hub.worldpop.org/project/categories?id=8), which provides geospatial population datasets with global coverage. The use of WorldPop data allowed for the analysis of population attributes at an aggregated level, ensuring that the study did not involve direct human subject participation.
Reporting on race, ethnicity, or other socially relevant groupings	This study did not involve the categorization and analysis of racial, ethnic, or other socially relevant groups.
Population characteristics	In this research, we categorized the population into three distinct age groups to facilitate a nuanced analysis of demographic patterns. These groups were defined as minors (ages 0-20 years), adults (ages 20-65 years), and elders (ages over 65 years). The data for this analysis was sourced from WorldPop (https://hub.worldpop.org/project/categories?id=8).
Recruitment	Participants were not recruited for this study.
Ethics oversight	This study was conducted using publicly available datasets and did not involve the collection of individual-level data. Given that the research solely relied on the analysis of existing public data without direct human participant involvement, it was not subject to review by an ethics committee in accordance with relevant guidelines and regulations. We ensured that all research activities adhered to applicable data protection laws and privacy principles, respecting the terms of use of the data sources.

Note that full information on the approval of the study protocol must also be provided in the manuscript.

Field-specific reporting

Please select the one below that is the best fit for your research. If you are not sure, read the appropriate sections before making your selection.

☐ Life sciences ☐ Behavioural & social sciences ☒ Ecological, evolutionary & environmental sciences

For a reference copy of the document with all sections, see nature.com/documents/nr-reporting-summary-flat.pdf

Ecological, evolutionary & environmental sciences study design

All studies must disclose on these points even when the disclosure is negative.

Study description	This study investigates the nonlinear impact of hydrological intrusion risks on migration dynamics across a comprehensive dataset of 46,776 global subnational units. Recognizing the complexity of factors influencing migration, we specifically aimed to uncover how varying degrees of water-related risks affect population movements at a granular geographic level. To achieve this, we employed factorial analysis methods. This method facilitated a nuanced understanding of how different factors combine and contribute to migration trends.
Research sample	The study presents an extensive investigation into global population migration patterns, covering an expansive dataset from 249 countries and regions, which includes a total of 46,776 subnational administrative units, primarily at the municipal or county level. To understand the multifaceted nature of migration, our research integrates a variety of multivariate variables, encompassing social, economic, geographic, and climatic factors. The data underpinning our analysis is sourced from several reputable databases, ensuring a robust and reliable foundation for our findings. Relevant data can be accessed through the following links: WorldPop, https://hub.worldpop.org/; Global surface water dynamics, https://glad.umd.edu/dataset/global-surface-water-dynamics; ERA5-Land, https://www.ecmwf.int/en/forecasts/dataset/ecmwf-reanalysis-v5; SRTM, https://www.earthdata.nasa.gov/sensors/srtm; NTL, https://dataverse.harvard.edu/dataset.xhtml?persistentId=doi:10.7910/DVN/YGIVCD; GDP, https://doi.org/10.1038/

s41597-022-01322-5; SHDI, https://globaldatalab.org/shdi/.

Sampling strategy	In this research, we utilized an extensive dataset that incorporates data from 46,776 subnational administrative units. Given the study's broad geographic coverage and its focus on a global-level analysis, traditional sample size calculations were not applicable.
Data collection	The dataset utilized in this study was collected from various open-source databases, ensuring a comprehensive and robust foundation for our analysis. The data acquisition and preparation process was a collaborative effort led by Dr. Renlu Qiao, Dr. Guobin Zhang, and Dr. Zhiyu Liu.
Timing and spatial scale	The data in this study are all annual, spanning from 2015 to 2020, and the spatial scales are all global.
Data exclusions	No data were excluded from the analyses.
Reproducibility	The study does not include the performance of any experimental procedures; instead, it relies on the application of sophisticated modeling techniques to analyze and interpret the available data.
Randomization	In the framework of the study, the sample was categorized based on distinct population characteristics, specifically gender and age, alongside the level of income as indicated by Gross National Income (GNI) per capita. To rigorously assess and control for the influence of various covariates on our findings, we employed a multivariate regression analysis technique. Furthermore, to address the possibility of unobserved heterogeneity and to ensure that our results are not biased by omitted variable factors that could vary across entities but remain constant over time, we incorporated fixed effects into our analysis.
Blinding	Given that this study utilized a non-interventional observational design, blinding was not applicable.

Did the study involve field work? ☐ Yes ☒ No

Reporting for specific materials, systems and methods

We require information from authors about some types of materials, experimental systems and methods used in many studies. Here, indicate whether each material, system or method listed is relevant to your study. If you are not sure if a list item applies to your research, read the appropriate section before selecting a response.

Materials & experimental systems

n/a	Involved in the study
☒	☐ Antibodies
☒	☐ Eukaryotic cell lines
☒	☐ Palaeontology and archaeology
☒	☐ Animals and other organisms
☒	☐ Clinical data
☒	☐ Dual use research of concern
☒	☐ Plants

Methods

n/a	Involved in the study
☒	☐ ChIP-seq
☒	☐ Flow cytometry
☒	☐ MRI-based neuroimaging

Plants

Seed stocks	This study is not relevant here.
Novel plant genotypes	This study is not relevant here.
Authentication	This study is not relevant here.
